# Effect of maternal body mass index on pregnancy outcome and newborn weight

**DOI:** 10.1186/1756-0500-5-34

**Published:** 2012-01-17

**Authors:** Shahla Yazdani, Yousofreza Yosofniyapasha, Bahman Hassan Nasab, Mohsen Haghshenas Mojaveri, Zinatossadat Bouzari

**Affiliations:** 1 Department of Obstetric & Gynecology of Babol University of Medical Sciences, Babol, Iran; 2 Department of Urology of Babol University of Medical Sciences, Babol, Iran; 3 Member of Infertility & Reproductive Health Research Center, Babol University of Medical Sciences, Babol, Iran; 4 Department of Anesthesia, Babol University of Medical Sciences, Babol, Iran; 5 Neonatologist, Non-Communicable Pediatrics Diseases Research Center, Babol University of Medical Sciences, Babol, Iran; 6 Member of Cellular & Molecular Biology Research Center, Babol University of Medical sciences, Babol Iran

## Abstract

**Background:**

Maternal obesity has been associated with adverse pregnancy outcomes, such as pre-eclampsia, eclampsia, pre- and post-term delivery, induction of labor, macrosomia, increased rate of caesarean section, and post-partum hemorrhage. The objective of this study was to determine the effect of maternal Body Mass Index (BMI) on pregnancy outcomes.

**Methods:**

1000 pregnant women were enrolled in the study. In order to explore the relationship between maternal first trimester Body Mass Index and pregnancy outcomes, participants were categorized into five groups based on their first trimester Body Mass Index. The data were analyzed using Pearson Chi-square tests in SPSS 18. Differences were considered significant if p < 0.05.

**Results:**

Women with an above-normal Body Mass Index had a higher incidence of pre-eclampsia, induction of labor, caesarean section, pre-term labor, and macrosomia than women with a normal Body Mass Index (controls). There was no significant difference in the incidence of post-term delivery between the control group and other groups.

**Conclusion:**

Increased BMI increases the incidence of induction of labor, caesarean section, pre-term labor and macrosomia. The BMI of women in the first trimester of pregnancy is associated with the risk of adverse pregnancy outcome.

## Background

The increasing incidence of obesity among women worldwide [1] has become one of the most significant public health concerns [2]. High maternal body mass index (BMI) is related to adverse maternal pregnancy outcomes such as pre-eclampsia, eclampsia, pre- and post-term delivery, induction of labor, macrosomia, caesarean section, and postpartum hemorrhage [3,4]. In 1990, the Institute of Medicine of the National Academies in the United States suggested that maternal weight gain during pregnancy should be based on pre-pregnancy BMI. For women with a BMI < 19.8 kg/m^2^, a weight gain of 28-40 lb was recommended; with a BMI of 19.8-24.9 kg/m^2^, a weight gain of 25-35 lb; with a BMI of 25.0-29.9 kg/m^2^, a weight gain of 15-25 lb; and finally with a BMI > 29.9 kg/m^2^, a weight gain of at least 15 lb with no uppert [5] is recommended. In 2006, attendees at the Institute of Medicine conference on the impact of pre-pregnancy weight on maternal and child health recommended that further research be done on the influence of weight gain in pregnancy [6].

Some researchers have agreed with the Institute of Medicine's initial recommendations for maternal weight gain during gestation [7-9]; however, recent studies suggest that lower gestational weight gain may be preferable [10,11]. In developing Asian countries, such as Iran, women generally have a lower BMI and/or a smaller gestational weight gain than in developed countries. In the USA, for example, 2% of pregnant women have a BMI < 18.5 and more than 50% have a BMI > 25 [12]. Hence, BMI seems to differ across populations. Taking this into account in combination with the possible effects of maternal BMI on pregnancy outcomes [13], there is a requirement to examine whether the current recommendations for pregnant women from the USA also apply to women from other countries such as Iran.

## Methods

This retrospective cohort study was conducted in the Yhyanejad Hospital of Babol University of Medical Sciences in Iran in 2008-2009. Among prime gravid women with a singleton uncomplicated pregnancy with cephalic presentation of 38 or more weeks of gestation, data were collected about height and weight during the first prenatal care after the positive pregnancy test was recorded. Then the BMI was calculated according to the formula weight/height^2^. All participant signed an informed consent approved by the Babol Medical Universities ethical committee. Data about the following pregnancy outcomes were collected during labour and birth: pre-eclampsia, induction of labour, caesarean section, preterm delivery, postdate delivery, and weight of newborn. These data were and written down in the chart by the resident in attendance after the positive pregnancy test during and after birth. We selected these pregnancy outcomes because they appear to be the most relevant pregnancy outcomes in relation to maternal BMI and health of mother and child.

Gestational age was calculated from the first day in the Last Menstrual Period (LMP) or taken from the dating ultrasound scan that was performed before 20 weeks of pregnancy. The BMI was calculated based on the formula weight/height^2^. Finally, we collected data about maternal age, drug abuse and smoking habit. Exclusions criteria were: abnormal fetal presentation, placenta previa, pre-eclampsia, gestational diabetes, medical disorders such as diabetes mellitus, chronic hypertension, cardiac or endocrine disorders and surgical conditions and unknown maternal weight in first trimester. The women were categorized into five sub groups according to their first trimester BMI as follows:

- Underweight: BMI ≤ 19.9 kg/m^2^

- Normal: BMI of 20-24.9 kg/m^2^

- Over weight: BMI of 25-29.9 kg/m^2^

- Obese: BMI of 30 -34.9 kg/m^2^

Morbid obese: BMI > 35 kg/m^2^

The group with normal BMI (20 - 24.9 kg/m^2^) was used as reference group for the analysis [2]. Optimum weight gain in pregnancy is based on this protocol:

-For BMI less than normal: 12-18.5 kg

-For normal BMI: 11.5-16 kg

-For more than normal: BMI equal or greater than 7-11.5 kg [14].

-For more than normal: BMI equal or greater than 7-11.5 kg [14].

### Statistical analysis

All data were analyzed by SPSS 18. Pearson Chi-square test was used to test the differences between the five categorized BMI groups regarding pregnancy outcomes and for gestational weight gain. We used the McNemar test to find out whether the groups were independent. A *p*-level < .05 was considered to be significant.

## Results

A total of 1356 pregnant women were enrolled in our study in 2008-2009. After applying the inclusion and exclusion criteria, the study sample consisted of 1000 pregnant women. Among these 1000 pregnant women;128 cases (12.8%) had BMI < 19, 9, 412 cases (41.2%) had a BMI 20-24.9, 356 cases (35.6%) had a BMI 25-29.9, 98 cases (9.8%) had a BMI 30-34.9 and 6 cases (0.6%) had a BMI ≥ 35. These women were categorized in BMI lower than normal, normal (reference group), overweight, obese, and morbid obese. Table 1 shows the results of the chi-square test by which pregnancy outcomes were compared between the 5 BMI-groups. A comparison of newborn weight based on maternal BMI in the first trimester of pregnancy in five groups (cases-control) is shown in Table 2.

**Table 1:** Chi-square tests of BMI- categories by pregnancy out comes.

BMI	20 >	20-24.9	25 - 29.9	30 - 34.9	35 <	*P*-value of chi-square test
Preeclampsia	5	19	31	12	2	0.001**
	3.9%	4.6%	8.7%	12.2%	3.33%	
Induction of labour	26	98	103	39	3	0.003**
	20.3%	23.8%	28.9%	40.9%	50%	
Caesarean section	11	59	71	25	4	0.001**
	8.6%	14.3%	19.9%	25.5%	7.66%	
Preterm delivery	2	9	16	6	1	0.037*
	1.6%	22.2%	4.7%	6.1%	0%	
Post date delivery	4	14	12	3	0	0.99
	3.1%	3.4%	2.4%	3%	0%	
Wound Infection	0	10	8	6	1	0.009**
		44%	32%	24%	4%	

** *p *< .01; * *p*- < .05

**Table 2:** Chi-square tests of newborn weight by maternal BMI in five categories

BMI	Birth Weight			*p*- value of chi-square test
	**2500 ≥**	**2500 - 4000**	**4000 ≤**	

20 >	23	105	0	0.001**
	18%	8.3%	0%	
20-24.9	28	370	14	0.001
	6.8%	89.8%	3.4%	
25-29.9	34	311	11	0.372
	9.6%	78.5%	2.9%	
30 - 34.9	9	79	10	0.01**
	9.2%	80.6%	10.2%	
> 35	0	5	1	0.01**
		83.4%	16.6%	

** *p *< .01; * *p*- < .05

Tables 1 and 2 shows that the incidence of pre-eclampsia increased with BMI and demonstrates that in nulliparous women the chance on caesarean section increased with BMI. Also, a comparison of induction of labour incases and reference groups showed that lower BMI is associated with lower induction of labour. In addition, BMI below normal is associated with a low birth rate, while aa BMI > 30 increases the chance on a higher birth weight (2500-4000 or higher).

For the evaluation of gestational weight gain during pregnancy on pregnancy outcomes and newborn weight in different BMI, we categorized the pregnant women into three groups based on their BMI: below normal, normal, and above normal (BMI < 20 kg/m^2^, BMI = 20-24.9 kg/m^2^, BMI > = 25 kg/m^2^, respectively). For the comparison of gestational weight gain we used the Mc Nemar test. A comparison of newborn weight based on maternal BMI during pregnancy is shown in Table 3.

**Table 3:** Comparison of newborn weight based on maternal BMI during pregnancy

BMI of the first trimester	Newborn weight		
	
	< 2500 gr	2500-4000 gr	4000 < gr
< 20	36(81.8%)	8(18.2%)	0(0%)
20-24.9(N)	48(5.2%)	839(90.3%)	42(4.5%)
25 ≤	10(37%)	9(33.3%)	8(29.6%)

In the group of women whose weight gain during pregnancy was below normal, 12.7% (7) had a first trimester BMI below normal, 54.5% (30) had a first trimester BMI within the normal range, and 32.7% (18) had a first trimester BMI above normal.

In the group of women whose weight gain during pregnancy was normal, 13.5% (118) had a first trimester BMI below normal, 37.5% (328) had a first trimester BMI within the normal range and 49% (429) had a first trimester BMI above normal.

In the group of women whose weight gain during pregnancy was above normal finally, 4.3% (3) had a first trimester BMI below normal, 77.1% (54) had a first trimester BMI within the normal range and 18.6% (13) had a first trimester BMI below normal.

## Discussion

This study shows that the incidence of preeclampsia increased with BMI. Robinson [15] and Leonie [16] showed in two separate studies that obese women are at high risk for pre-eclampsia which is in line with the results of this study. This study demonstrated that in nulliparous women the chance on caesarean section increased with BMI, this result is similar to findings of Hoff [17] and Berghof [3]. Comparison of induction of labour incases and reference groups showed that lower BMI was associated with lower induction of labour. This is similar to results of Ushakiran [18] and Heather [13]. We found that nulliparous women with a higher BMI had a higher percent of pre term labour, which is similar to previous studies from Bhattacharya [2] and Callaway [14]. Although for post-date delivery there was no difference between cases and control groups in this study like Seligman [19], Ushakiran and collegues [18] found that post-date delivery increased in women with BMI > 30. BMI in the first trimester was related to birth weight and maximum rate of macrosomic was found in obese/morbid obese group and macrosomic was minimal in BMI < normal. The latter result is similar to other studies from Ushakiran [18] and Bhattacharya [20] for instance.

## Conclusion

This research demonstrates that maternal BMI is an important risk factor of pre-eclampsia. An increased BMI increases the incidence of induction of labour, caesarean section, pre term labour and macrosomia. Therefore, we advice pregnant woman to gaina normal BMI of 20-24 [2], before and during pregnancy, for instance by consulting their physician or a dietitian prior to getting pregnant.

## Study Limitations

The present study had several limitations. For example, this study was performed in 2 hospitals in Babol city and does not cover all Iran hospitals, which may affect the external validity of the findings. In addition, the sample was not homogeneous with regard to age, education and socio-economic status. All these factors may impact quality of life and BMI and, hence, the study results. We suggest to future researchers to consider other effective factors besides BMI such as age, education, economic status and quality of life on pregnancy outcomes.

## Abbreviations

(BMI): Body Mass Index.

## Competing interests

The authors declare that they have no competing interests.

## Authors' contributions

Each author has participated actively and sufficiently in this study. ZB conceived the idea and design of the study, interpretation of data, and drafted the manuscript. SHY conceived the idea and design of the study. ZSH and MH made a substantial contribution to analysis and interpretation of data. BHN, and MM have contributed to the data collection and the editing of the manuscript. Each author revised the manuscript and provided final approval of the version to be published.
